# Serum long non-coding RNA SCARNA10 serves as a potential diagnostic biomarker for hepatocellular carcinoma

**DOI:** 10.1186/s12885-022-09530-3

**Published:** 2022-04-20

**Authors:** Yawei Han, Wenna Jiang, Yu Wang, Meng Zhao, Yueguo Li, Li Ren

**Affiliations:** 1 Department of Clinical Laboratory, Tianjin Medical University Cancer Institute and Hospital, National Clinical Research Center for Cancer, Key Laboratory of Cancer Prevention and Therapy, Tianjin’s Clinical Research Center for Cancer, Huanhuxi Road, Hexi District, Tianjin, 300060 China; 2 College of Inspection, Tianjin Medical University, Tianjin, China

**Keywords:** Hepatocellular carcinoma, Long non-coding RNA, SCARNA10, Biomarker

## Abstract

**Background:**

Circulating long non-coding RNAs (lncRNAs) have been demonstrated to serve as diagnostic or prognosis biomarkers for various disease. We aimed to elucidate the diagnostic efficacy of serum lncRNA SCARNA10 for the hepatocellular carcinoma (HCC).

**Methods:**

In this study, a total of 182 patients with HCC, 105 patients with benign liver disease (BLD), and 149 healthy controls (HC) were enrolled. According to different classifications, the levels of serum SCARNA10 were assessed by quantitative real-time polymerase chain reaction (qPCR). The correlations between serum SCARNA10 and clinicopathological characteristics were further analyzed. The receiver operating characteristic (ROC) curve and area under curve (AUC) were utilized to estimate the diagnostic capacity of serum SCARNA10 and its combination with AFP for HCC.

**Results:**

The results demonstrated that the levels of serum SCARNA10 were significantly higher in HCC patients than in patients with BLD and healthy controls, and significantly increased in HCC patients with hepatitis B or C infection, or with liver cirrhosis. Furthermore, positive correlations were noted between serum SCARNA10 level and some clinicopathological characteristics, including tumor size, differentiation degrees, tumor stage, vascular invasion, tumor metastasis and complications. ROC analysis revealed that SCARNA10 had a significantly predictive value for HCC (Sensitivity = 0.70, Specificity = 0.77, and AUC = 0.82), the combination of SCARNA10 and AFP gained the higher sensitivity (AUC_SCARNA10 + AFP_ = 0.92 vs AUC_AFP_ = 0.83, *p* <  0.01). SCARNA10 retained significant diagnosis capabilities for AFP-negative HCC patients.

**Conclusions:**

In summary, lncRNA SCARNA10 may serve as a novel and non-invasive biomarker with relatively high sensitivity and specificity for HCC diagnosis.

**Supplementary Information:**

The online version contains supplementary material available at 10.1186/s12885-022-09530-3.

## Introduction

Hepatocellular carcinoma (HCC) is one of the most common malignant tumors globally with relatively high morbidity and mortality [1, 2]. Although great progresses made in HCC therapy in recent years, the prognosis of HCC patients still remains poor due to being diagnosed at an advanced stage and high rate of recurrence [1, 3]. Currently, histological examination of the tumor tissue serves as the standard procedure for definitive diagnosis of HCC, and imaging examinations including computer tomography (CT) and magnetic resonance imaging (MRI) are the common supplement procedures [4]. However, their application has been limited due to invasiveness, insensitivity to small tumors, and high cost [5]. For serum biomarkers, alpha fetoprotein (AFP) is one of the most widely used biomarkers for HCC diagnosis clinically. Nevertheless, the sensitivity and specificity of AFP are unsatisfactory, especially for patients with early-stage HCC. In addition, AFP may be elevated in some benign liver diseases, such as chronic hepatitis and cirrhosis [6, 7]. Therefore, it is of great value to detect convenient, non-invasive, inexpensive and repeatable serum biomarkers in the HCC diagnosis.

Long non-coding RNAs (lncRNAs) are a class of non-coding RNA, which is longer than 200 nucleotides in length [8]. Accumulating evidence has demonstrated that lncRNAs play important roles in various physiological and pathological processes [9, 10]. LncRNAs have been proved to play important roles as oncogenes or tumor suppressor genes in different cancer types [11]. Current researchers have found that lncRNA abnormal expression profile has close relationships with HCC developments [12]. Several circulating lncRNAs have been implied to be promising markers with high accuracies and efficiencies for the diagnosis and prognosis of HCC, for instance, HULC, MALAT1 and UCA1 [13–17]. However, the diagnostic value of many lncRNAs for HCC reported by different researchers remains controversial and needs further investigation. Moreover, the diagnostic efficacy of only a small number of circulating lncRNAs has been confirmed. The majority of circulating lncRNAs involved in HCC still remains largely unclear.

Previously, we identified lncRNA Small Cajal body-specific RNA 10 (SCARNA10) is up-regulated in the serum and liver tissue samples from patients with advanced hepatic fibrosis, which promotes liver fibrosis both in vitro and in vivo through inducing HCs apoptosis and HSCs activation [18]. It has been reported that SCARNA10 is one of the highest expressed in 615 differentially expressed lncRNAs of breast cancer [19]. SCARNA10 has also been reported to play important potential functions in small-cell lung cancer (SCLC) [20]. Although the function of SCARNA10 in liver tissues are beginning to be understood, its roles in serum of HCC patients require further study.

Here, we initially found that serum SCARNA10 level is higher in HCC than in healthy controls, SCARNA10 level was correlated with tumor differentiation degrees, size, stage, vascular invasion, lymph node metastasis, distant metastasis and complications. Combined detection of SCARNA10 and AFP was more effective for the diagnosis of HCC. SCARNA10 may serve as a potential diagnostic biomarker for HCC.

## Methods and materials

### Patients and specimens

This study analyzed a total of 281 serum samples obtained from 127 patients diagnosed with HCC, 55 patients diagnosed with benign liver disease (BLD) and 99 healthy controls (HC) at Tianjin Medical University Cancer Institute and Hospital (Tianjin, China) between October 2019 and March 2021. In addition, we collected 50 patients diagnosed with HCC, 50 patients with BLD and 50 HCs at Tianjin Medical University General Hospital for external validation. Patients with hemorrhagic or thrombotic diseases and those who have taken anticoagulant agents such as warfarin and similar drugs or vitamin K within 6 months of enrollment were excluded. The baseline characteristics of HCC patients, BLD patients and HCs were summarized in Table 1 and Supplementary Table 1.

**Table 1:** Characteristics of HC, patients with BLD and HCC

		HC (*n* = 99) Number (%)	BLD (*n* = 55) Number (%)	HCC (*n* = 127) Number (%)
Age (median [IQR])	years	53 (42–59)	50 (39–59)	56 (45–65)
Gender (%)	Male	48 (48.48)	30 (54.55)	81 (63.78)
	Female	51 (51.52)	25 (45.45)	46 (36.22)
HBV antigen (%)	Negative	99 (100.00)	32 (58.18)	77 (60.63)
	Positive	0 (0)	23 (41.82)	50 (39.37)
HCV antibody (%)	Negative	99 (100.00)	38 (69.09)	95 (74.80)
	Positive	0 (0)	17 (30.91)	32 (25.20)
Pathology (%)	Hepatitis	0 (0)	40 (72.73)	64 (50.39)
	Cirrhosis	0 (0)	15 (27.27)	63 (49.61)
	HCC	0 (0)	0 (0)	127 (100)

Venous blood were collected from the participants before treatment, centrifuged at 3000 rpm for 20 min, transferred the supernatant to EP tube, and quickly frozen to − 80 °C for later use, avoiding freezing and thawing cycles. Clinicopathological feature data including age, sex, tumor size and number, stage, lymph node and distant metastasis, vascular invasion, differentiation degree and complicatons were collected.

### RNA extraction and qPCR

Total RNA was extracted from equal volume of serum samples with TRIzol LS reagent (Invitrogen, Carlsbad, CA, USA) as described in the manufacturer’s protocol. All RNAs were digested with DNase I (Takara, Dalian, China). cDNA was generated using the First-strand cDNA synthesize kit (#1622, Thermo Fisher Scientific, Waltham, MA, USA) with random hexamer primers in accordance with the manufacturer’s instructions. The synthesized cDNA templates were further amplified by SYBR Green master kit (Takara, Dalian, China), and the expression of β-actin was used as the internal control. We calculated the relative expression values by comparing the normalized cycle threshold (Ct). The sequences of primers were as follows: SCARNA10 (forward): 5′-GTTTGGCTAAGCCCAGGGAC-3′, (reverse): 5′-GTTGGTCTGCCCTTACAGTGA-3′; β-actin (forward): 5′-GCCGGGACCTGACTGACTAC-3′, (reverse): 5′-TTCTCCTTAATGTCACGCACGAT-3′.

### Quantification of serum AFP levels using electrochemiluminescence immunoassays

Serum AFP levels were measured using the Roche Cobas E601 electrochemical immune luminescence analyzer (Roche Diagnostics, Mannheim, Germany) equipped with Roche dedicated reagents following the instructions provided by the manufacturer.

### Statistical analysis

The date was analyzed by the Mann-Whitney test to assess differences between groups. Spearman’s correlation test was used to determine the relationship between serum SCARNA10 levels and biochemical parameters. The ROC analysis was performed to obtain the area under the curve (AUC), cut-off values, sensitivity, specificity, positive predictive value (PPV) and negative predictive value (NPV). Z-test was used to compare the AUCs between two groups, and ANOVA with post hoc analysis was used for the comparison of three groups. *P*-value < 0.05 was considered to indicate a significant difference. All statistical analyses were performed using SPSS 20.0 (IBM, Armonk, NY, USA).

## Results

### The expression level of lncRNA SCARNA10 in serum of HCC patients

**Fig. 1:**
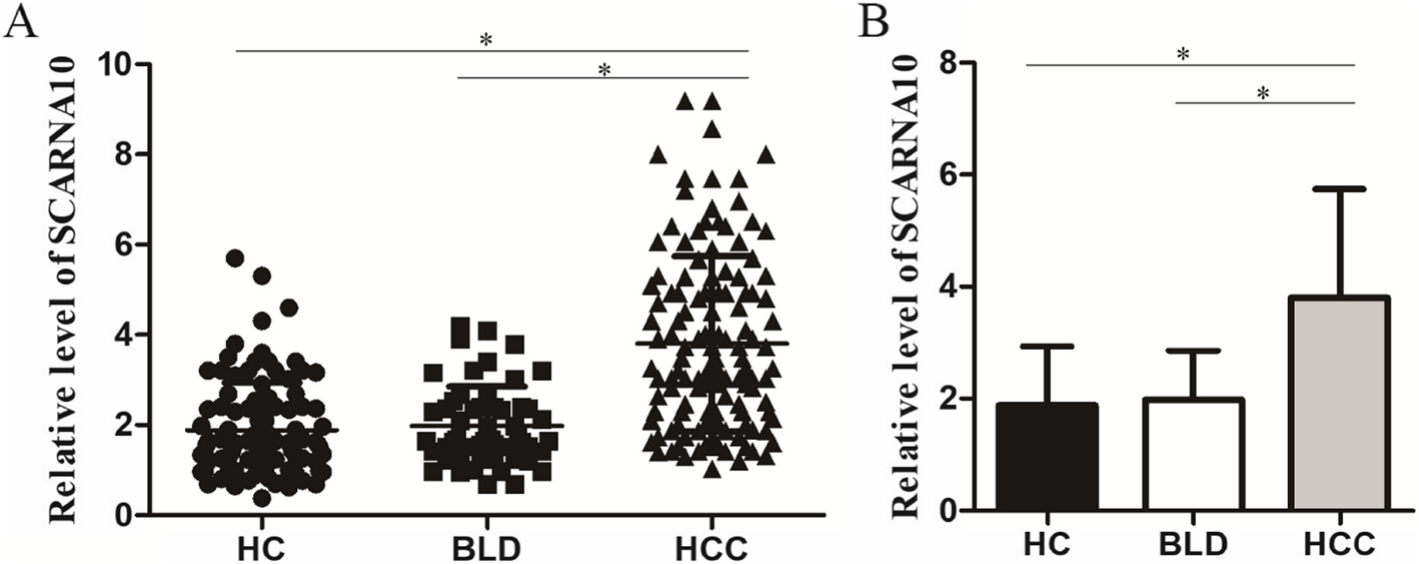
Serum SCARNA10 levels in participants. **A**, **B** The relative levels of SCARNA10 in patients with HCC, benign liver diseases (BLD) and healthy controls (HC) were performed by qPCR. Scatter plot (**A**) and bar plot (**B**) are shown for SCARNA10 in HCC, BLD and HC. *p <  0.05

To determine the significance of lncRNA SCARNA10 in HCC, the expression levels of SCARNA10 in serum of 127 HCC patients, 55 benign liver disease (BLD) and 99 healthy controls (HC) were examined by qPCR analysis. The relative serum levels of SCARNA10 in HCC patients were significantly higher than that in patients diagnosed with BLD and HC (both *p* <  0.05, Fig. 1). Simultaneously, we observed that SCARNA10 expression levels were not significantly difference between patients with BLD and HC (Fig. 1). Furthermore, we verified the level of SCARNA10 in external samples, including 50 patients with HCC, 50 patients with BLD and 50 HCs, and the results also showed that the level of SCARNA10 was increased in the serum of patients with HCC (Supplementary Figure 1). These results suggested that SCARNA10 can be used as a potential diagnostic biomarker for HCC.

### Subgroup analysis of serum SCARNA10 level in HCC patients

**Fig. 2:**
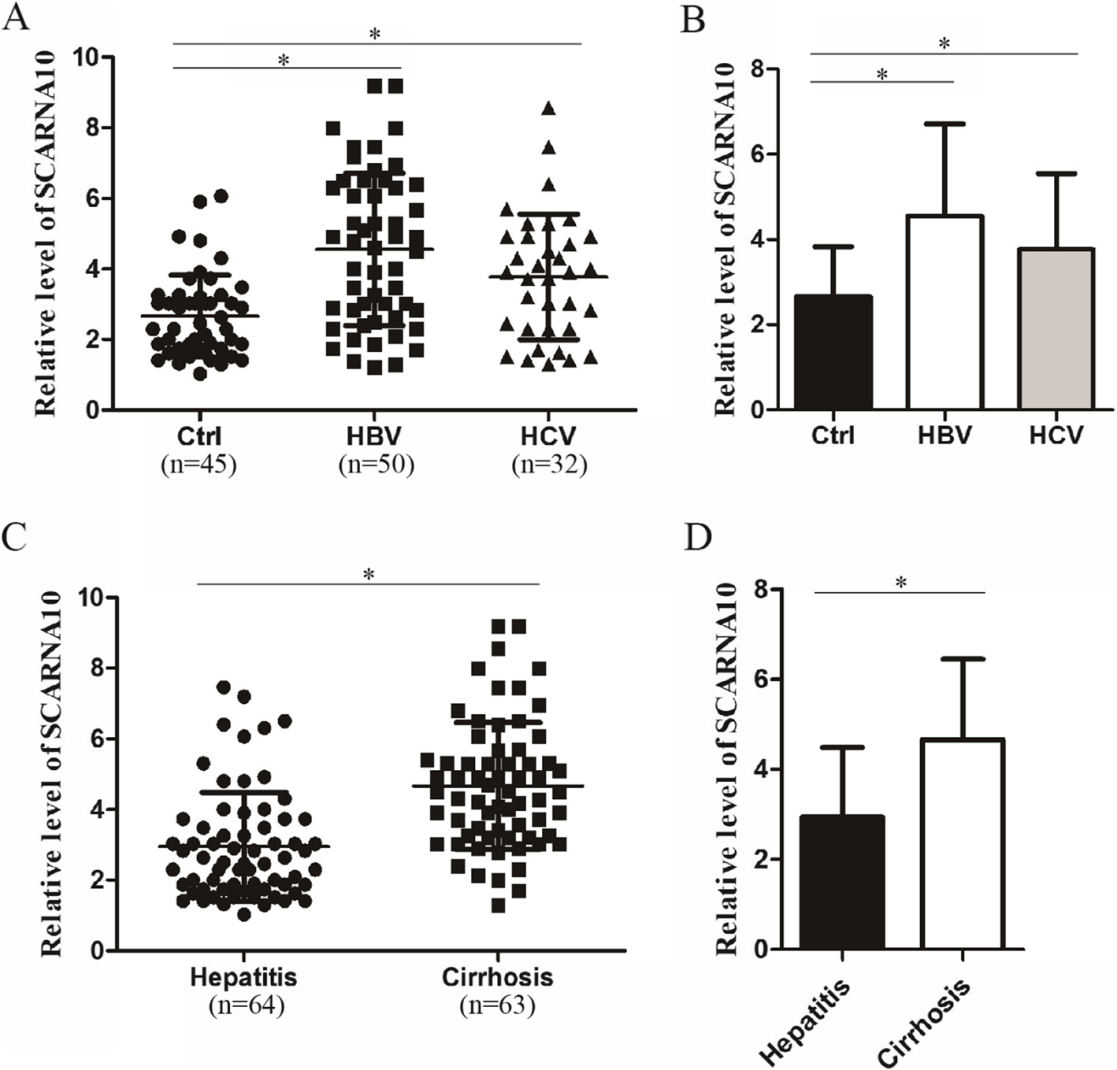
Serum SCARNA10 levels in patients with HCC. **A**, **B** Serum SCARNA10 levels in HCC patients with hepatitis B virus (HBV) infection, hepatitis C virus (HCV) infection, or virus infection with neither (Ctrl). Scatter plot (**A**) and bar plot (**B**) are shown for SCARNA10. **C**, **D** Serum SCARNA10 levels in HCC patients with chronic hepatitis or liver cirrhosis were performed by qPCR. Scatter plot (**C**) and bar plot (**D**) are shown for SCARNA10. *p <  0.05

According to different classification methods, we further found that serum SCARNA10 levels in HCC patients with hepatitis B virus or hepatitis C virus infection were significantly higher than those without virus infection controls (both *p* <  0.05, Fig. 2A-B), but there was no significant difference in the expression of SCARNA10 between HCC patients infected with HBV and HCV (Fig. 2A-B). Moreover, serum SCARNA10 levels in HCC patients with liver cirrhosis were significantly increased (*p* <  0.05, Fig. 2C-D).

### Association between clinicopathological features and SCARNA10 expression levels in HCC

To investigate the clinical significance of SCARNA10 expression in HCC, the relationship between serum SCARNA10 levels and clinicopathological features in HCC patients were summarized in Table 2. We found that SCARNA10 expression was significantly correlated with tumor size and differentiation degrees (both *p* <  0.01). Moreover, serum SCARNA10 levels was positively correlated with the tumor stage (*p* <  0.01). Serum SCARNA10 levels in HCC patients with vascular invasion, lymph node metastasis and distant metastasis were significantly increased (all *p* < 0.05). Simultaneously, serum SCARNA10 levels in patients experiencing complications were significantly higher than the levels observed in patients without complications (*p* < 0.01). However, there was no significant correlation between the SCARNA10 levels and age, sex, or tumor numbers.

**Table 2:** Correlation of serum SCARNA10 levels with clinipathological parameters in HCC

Characteristics	Number (%)	Serum SCARNA10 levels		***p***-value
		Low levels ≤ 3.25 (*n* = 65) Number (%)	High levels > 3.25 (*n* = 62) Number (%)	
Age (years)				
≤ 55	58 (45.67)	27 (21.26)	31 (24.41)	0.437
> 55	69 (54.33)	38 (29.92)	31 (24.41)	
Gender				
Male	81 (63.78)	41 (32.28)	40 (31.50)	0.297
Female	46 (36.22)	24 (18.90)	22 (17.32)	
Tumor size				
≤ 5 cm	76 (59.84)	47 (37.01)	29 (22.83)	< 0.01^*^
> 5 cm	51 (40.16)	18 (14.17)	33 (25.98)	
Tumor number				
Single	79 (62.20)	40 (31.50)	39 (30.71)	0.495
Multiple	48 (37.80)	25 (19.69)	23 (18.11)	
Differentiation degrees				
Well	48 (37.80)	34 (26.77)	14 (11.02)	< 0.01^*^
Moderate	46 (36.22)	19 (14.96)	27 (21.26)	
Poor	33 (25.98)	12 (9.45)	21 (16.54)	
Tumor stage				
1	49 (38.58)	33 (25.98)	16 (12.60)	< 0.01^*^
2	49 (38.58)	23 (18.11)	26 (20.47)	
3	19 (14.96)	6 (4.72)	13 (10.24)	
4	10 (7.88)	3 (2.36)	7 (5.51)	
Vascular invasion				
Yes	58 (45.67)	28 (22.05)	30 (23.62)	< 0.01^*^
No	69 (54.33)	37 (29.13)	32 (25.20)	
Lymph node metastasis				
Yes	45 (35.43)	19 (14.96)	26 (20.47)	< 0.01^*^
No	82 (64.57)	46 (36.22)	36 (28.35)	
Distant metastasis				
Yes	33 (25.98)	18 (14.17)	15 (11.81)	0.038^*^
No	94 (74.02)	47 (37.01)	47 (37.01)	
Complications				
Yes	47 (37.01)	20 (15.75)	27 (21.26)	< 0.01^*^
No	80 (62.99)	45 (35.43)	35 (27.56)	

### The diagnostic value of SCARNA10 in HCC patients

**Fig. 3:**
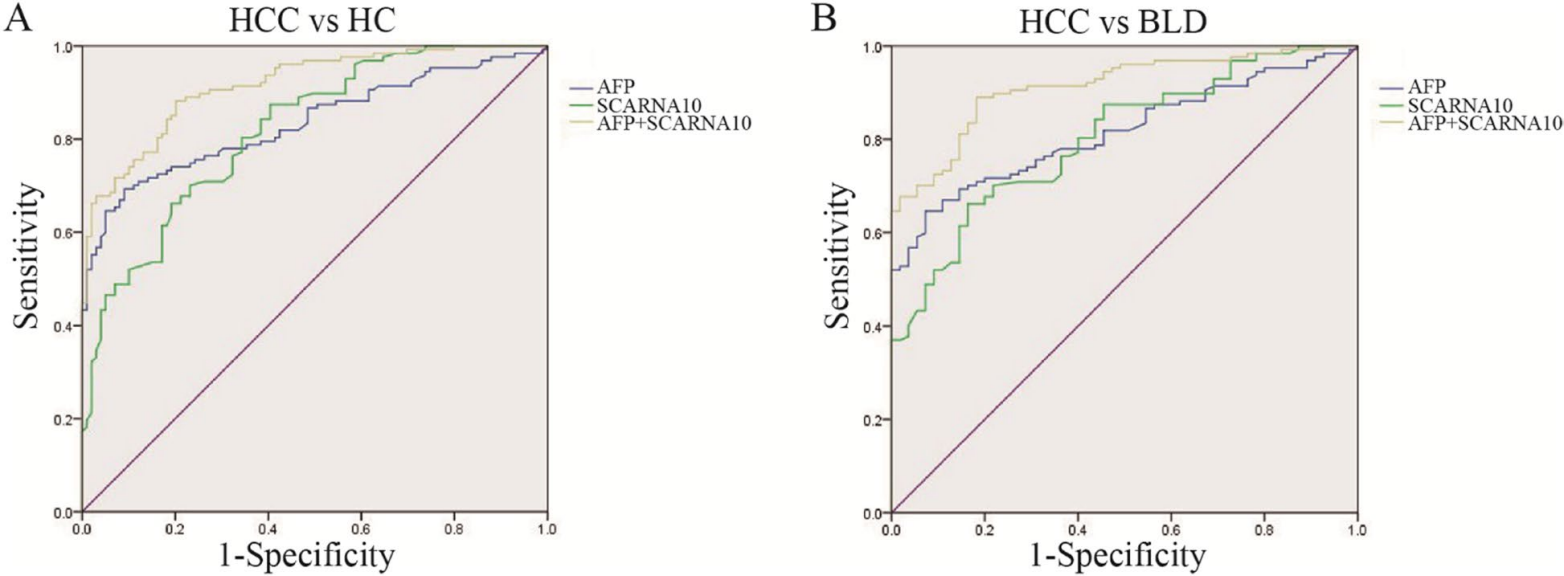
SCARNA10 and AFP complementation in the diagnosis of HCC. ROC of SCARNA10, AFP, SCARNA10 + AFP to distinguish HCC from HC (**A**), and BLD (**B**)

To further explore the utility of SCARNA10 as a promising diagnostic molecular marker for HCC, ROC curves of SCARNA10 and AFP were plotted in participants. As shown in Fig. 3 and Table 3, both markers showed remarkable diagnostic performance in distinguishing HCC from HC, and both markers showed remarkable diagnostic performance when it came to distinguishing HCC from BLD. The AUCs of the combined markers were significantly greater than SCARNA10 or AFP alone in all groups. The sensitivity of the diagnosis of the two combined markers were also increased, respectively. Additionally, we plotted ROC curves of SCARNA10 and AFP in participants of external validation (Supplementary Figure 2). The AUCs, sensitivity, specificity and cut-off were also showed in supplementary Table 2. These results of the external verification are consistent with the main text.

**Table 3:** Performances of SCARNA10 and AFP for the diagnosis of HCC patients

	Sensitivity	Specificity	Cut-off	PPV	NPV	AUC	z-test	***p***-value
**HCC vs HC**								
SCARNA10	0.70	0.77	2.83 (2^-△△Ct^)	0.82	0.65	0.82^*^	5.143	< 0.01
AFP	0.69	0.91	12.40 (ng/ml)	0.97	0.63	0.83^*^	3.255	< 0.01
SCARNA10 + AFP	0.88	0.80				0.92		
**HCC vs BLD**								
SCARNA10	0.66	0.84	1.74 (2^-△△Ct^)	0.82	0.63	0.80^*^	4.847	< 0.01
AFP	0.67	0.89	31.20 (ng/ml)	1.00	0.45	0.82^*^	3.588	< 0.01
SCARNA10 + AFP	0.89	0.82				0.91		

*p < 0.01 in comparison with SCARNA10 + AFP

### Efficacy of SCARNA10 in the diagnosis of AFP-negative HCC patients

**Fig. 4:**
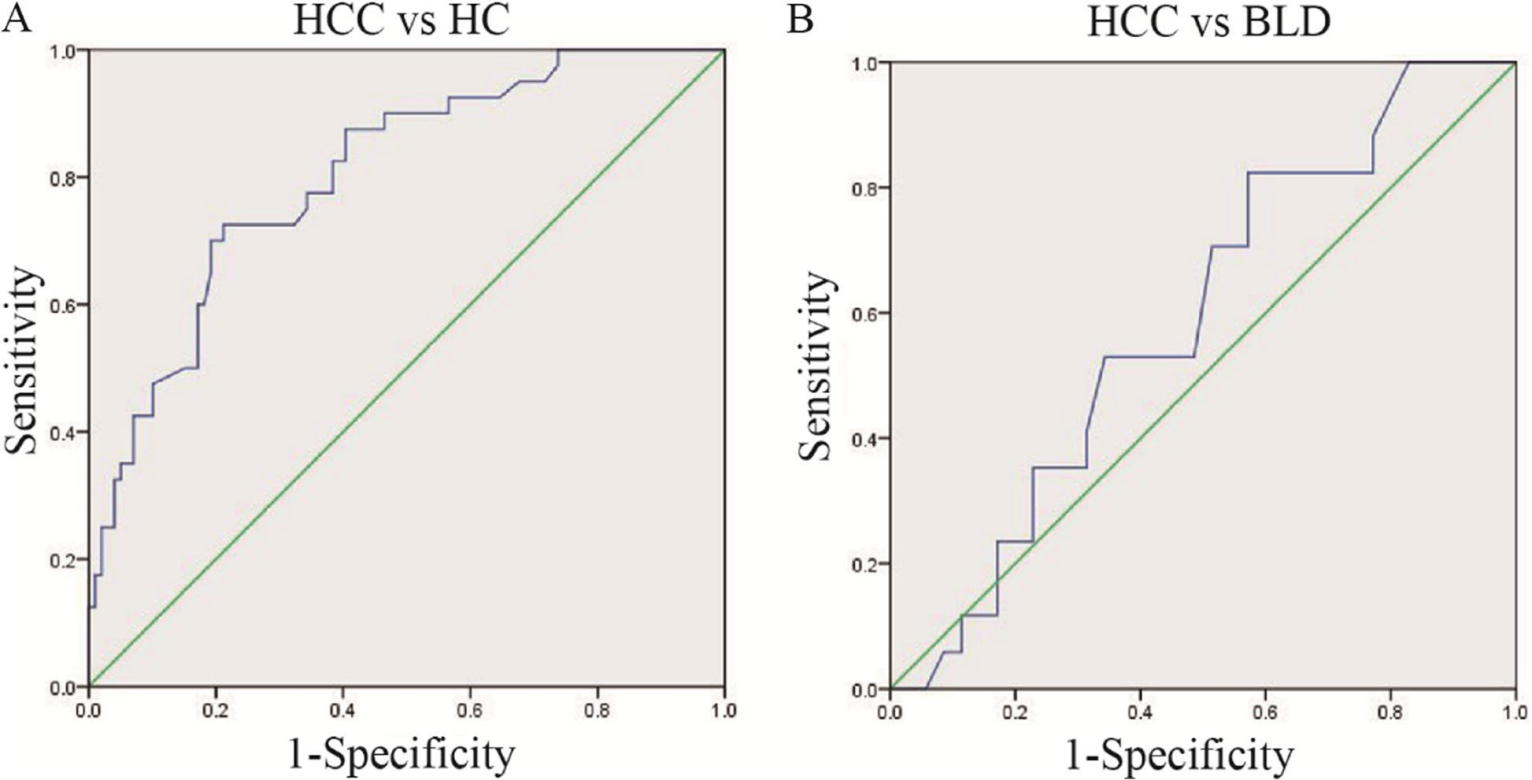
Performance of SCARNA10 in the diagnosis of AFP-negative HCC patients. ROC of SCARNA10 to distinguish HCC AFP-negative from HC (**A**), and BLD (**B**), respectively

To further estimate the complementary role of SCARNA10 for AFP in the diagnosis of HCC, the diagnostic value of SCARNA10 were assessed in HCC patients that were missed by AFP, based on the cut-off values obtained in this study (12.40 ng/ml and 31.20 ng/ml). As shown in Fig. 4 and Table 4, SCARNA10 showed a significant ability in distinguishing AFP-negative HCC from healthy controls with higher sensitivity and specificity.

**Table 4:** Performance of SCARNA10 for the diagnosis of AFP-negative HCC patients

	Sensitivity	Specificity	Cut-off	PPV	NPV	AUC
HCC vs HC	0.73	0.79	1.52 (2^-△△Ct^)	0.40	0.93	0.80
HCC vs BLD	0.82	0.43	2.83 (2^-△△Ct^)	0.76	0.79	0.59

## Discussion

Currently, the identification of novel potential serum biomarkers for the detection of HCC remains a vital goal, particularly for the diagnosis of early-stage HCC. Nevertheless, only a few biomarker candidates have been translated to clinical applications due to the limited study cohorts or diagnostic performance. For instance, so far, there is still only AFP as the most commonly used biomarker for HCC patients, despite its unsatisfactory sensitivity and specificity [7]. Several HCC-related lncRNAs can be present in the body fluid, as is the case lncRNAs UCA1, WRAP53, PVT1, ATB and uc002mbe.2 [17, 21–23]. The representative lncRNA HULC is detectable in blood sample and can be easily quantified by conventional qPCR [24]. These findings have suggested a non-invasive approach for HCC diagnosis through circulating lncRNA measurement. Our study is the first to examine the potential diagnostic utility of serum SCARNA10 in HCC patients.

As previously researches, a large number of lncRNAs are aberrantly expressed in HCC compared with normal liver tissue, which is useful to distinguish HCC patients from healthy cohorts [25]. However, some of those lncRNAs are also shown aberrant expression patterns in other cancer types or non-cancerous situations such as cirrhosis or liver injury, resulting in reduced reliability. Thus, lncRNAs combined with other molecules, especially known HCC biomarker AFP, is more likely to be a desirable HCC diagnosis method instead of evaluating lncRNA alone. For example, the combination of two lncRNAs UCA1 and WRAP53 with AFP achieves sensitivity up to 100% [21]. Similarly, the combination of another two lncRNAs PVT1 and uc002mbe.2 with AFP have been also shown to perform much better than AFP alone in HCC diagnosis [22]. Besides AFP, other molecules including miRNAs or mRNAs can also predict HCC in combination with lncRNAs [26]. In this study, for the first time, we found that the levels of SCARNA10 in HCC patients were significantly higher than that in BLD patients and healthy controls. Moreover, the SCARNA10 levels were not notable divergence between BLD patients and HC. These demonstrated that serum SCARNA10 levels can clearly distinguish benign and malignant liver tumors. In addition, the ROC analysis suggested that the AUCs of the combined SCARNA10 and AFP were greater than one alone in distinguishing HCC from BLD and HCC from HC. The sensitivity of the two combined markers were also increased. Finally, in AFP-negative HCC, SCARNA10 also showed a significant ability with higher sensitivity and specificity. All these findings indicated that SCARNA10 can serve as a potential diagnostic biomarker for HCC.

HBV infection is the major risk factor for HCC development. Worldwide, > 50% of HCC cases are associated with HBV infection [2, 27]. Several lines of evidence have supported the direct involvement of HBV in driving hepatocarcinogenesis. For example, the HBV genome can integrate into the human genome, contributing to genomic instability and generation of oncogenic chimeric transcripts [28]. HBV protein X (HBx) is highly carcinogenic, and 90% of HBx transgenic mice develop HCC [29]. HCV infection is a major cause of cirrhosis and consequently HCC [30]. The molecular mechanisms underlying HCV-induced HCC development might differ from those associated with HBV infection. HCV could promote HCC formation by upregulating host miRNAs and deregulating cellular signaling pathways [31, 32]. In this study, we found that serum SCARNA10 levels in HCC patients with HBV or HCV infection were significantly higher than those without infection controls, and in HCC patients with liver cirrhosis were also increased. These results demonstrated that SCARNA10 may play a role in HCC by regulating a common pathway in HBV or HCV. All these provide ideas for further research on the function and mechanism of SCARNA10 in HCC.

In conclusion, we found the serum SCARNA10 level was increased in HCC patients, and associated with some clinicopathological features, including tumor size, differentiation degrees, stage, vascular invasion, metastasis and complications. The combined detection of SCARNA10 and AFP significantly improved the diagnostic sensitivity of HCC. However, the roles of SCARNA10 in HCC need to be elucidated in more detail. In future study, we will further research the definite function and mechanism of SCARNA10 in liver carcinogenesis.

## Supplementary Information

**Additional file 1: Supplementary figure 1.** Serum SCARNA10 levels in participants of external validation. (A-B) The relative levels of SCARNA10 in patients with HCC, benign liver diseases (BLD) and healthy controls (HC) were performed by qPCR. Scatter plot (A) and bar plot (B) are shown for SCARNA10 in HCC, BLD and HC. *p < 0.05. **Supplementary figure 2.** SCARNA10 and AFP complementation in the diagnosis of HCC. ROC of SCARNA10, AFP, SCARNA10 + AFP to distinguish HCC from HC (A), and BLD (B). **Supplementary Table 1.** Characteristics of participants in external validation. **Supplementary Table 2.** Performances of SCARNA10 and AFP for the diagnosis of HCC patients in external validation.

## Data Availability

The datasets used during the current study are available from the corresponding author without restriction.

## Abbreviations

lncRNAs: Long non-coding RNAs
HCC: Hepatocellular carcinoma
BLD: Benign liver disease
HC: Healthy controls
qPCR: Quantitative real-time polymerase chain reaction
ROC: Receiver operating characteristic
AUC: Area under curve
CT: Computer tomography
MRI: Magnetic resonance imaging
AFP: Alpha fetoprotein
SCARNA10: Small Cajal body-specific RNA 10
