# Supplementary material for: Serum long non-coding RNA SCARNA10 serves as a potential diagnostic biomarker for hepatocellular carcinoma
Source: BMC Cancer. 2022 Apr 20;22:431. doi: 10.1186/s12885-022-09530-3 (PMC9022341; doi:10.1186/s12885-022-09530-3)
Supplement: Supplementary file 1 — Additional file 1: Supplementary figure 1. Serum SCARNA10 levels in participants of external validation. (A-B) The relative levels of SCARNA10 in patients with HCC, benign liver diseases (BLD) and healthy controls (HC) were performed by qPCR. Scatter plot (A) and bar plot (B) are shown for SCARNA10 in HCC, BLD and HC. *p < 0.05. Supplementary figure 2. SCARNA10 and AFP complementation in the diagnosis of HCC. ROC of SCARNA10, AFP, SCARNA10 + AFP to distinguish HCC from HC (A), and BLD (B). Supplementary Table 1. Characteristics of participants in external validation. Supplementary Table 2. Performances of SCARNA10 and AFP for the diagnosis of HCC patients in external validation. [file 12885_2022_9530_MOESM1_ESM.docx]

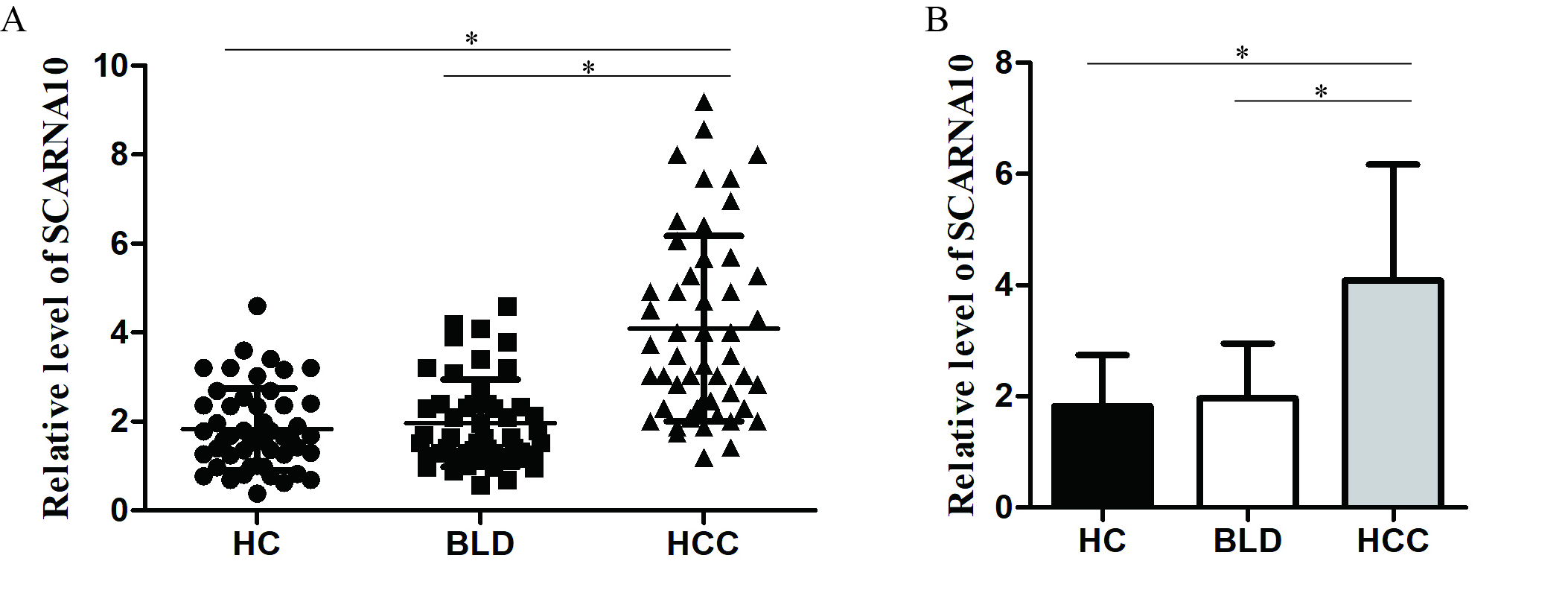


**Supplementary figure 1.** **Serum SCARNA10 levels in participants of external validation.** (A-B) The relative levels of SCARNA10 in patients with HCC, benign liver diseases (BLD) and healthy controls (HC) were performed by qPCR. Scatter plot (A) and bar plot (B) are shown for SCARNA10 in HCC, BLD and HC. **p*<0.05.


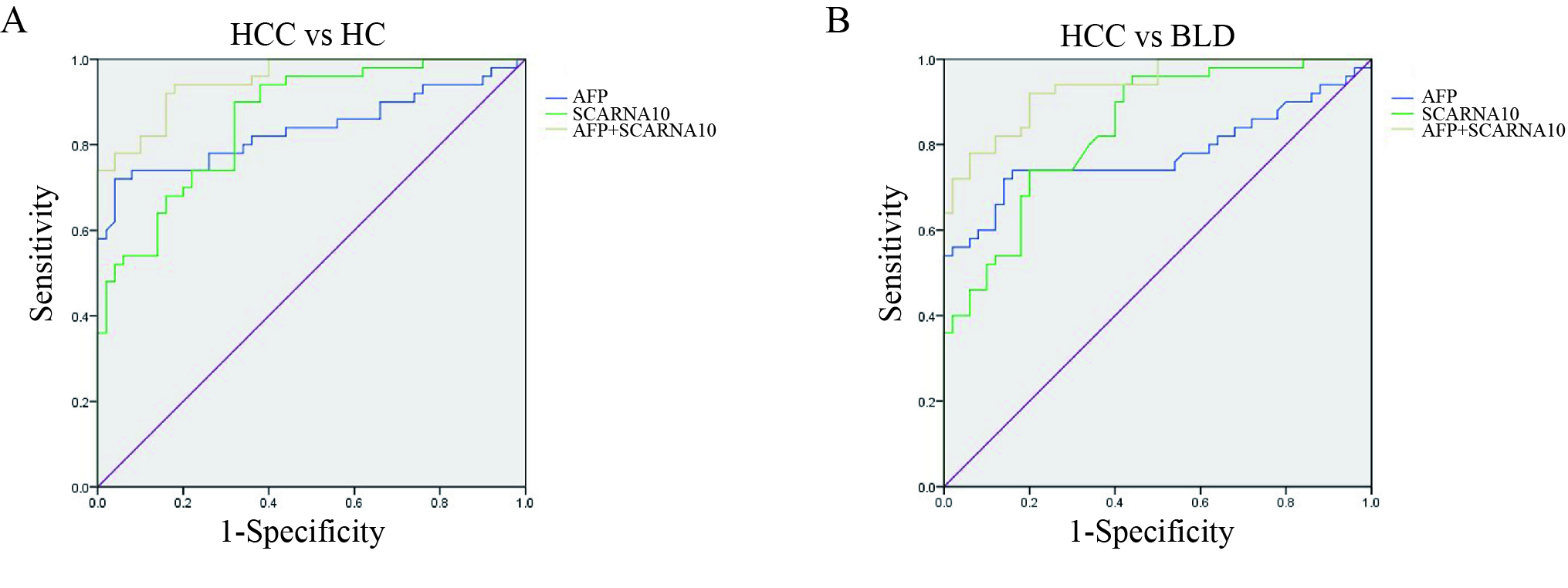


**Supplementary figure 2. SCARNA10 and AFP complementation in the diagnosis of HCC.** ROC of SCARNA10, AFP, SCARNA10 + AFP to distinguish HCC from HC (A), and BLD (B).

**Supplementary Table1** Characteristics of participants in external validation

|  |  | HC (n=50)  Number (%) | BLD (n=50)  Number (%) | HCC (n=50)  Number (%) |
| --- | --- | --- | --- | --- |
| Age (median [IQR]) | years | 51 (41-60) | 55 (45-60) | 53 (41-63) |
| Gender (%) | Male | 26 (52.00) | 28 (56.00) | 30 (60.00) |
|  | Female | 24 (48.00) | 22 (44.00) | 20 (40.00) |

**Supplementary Table2** Performances of SCARNA10 and AFP for the diagnosis of HCC patients in external validation

|  | **Sensitivity** | **Specificity** | **Cut-off** | **PPV** | **NPV** | **AUC** | **z-test** | ***p*-value** |
| --- | --- | --- | --- | --- | --- | --- | --- | --- |
| **HCC vs HC** |  |  |  |  |  |  |  |  |
| SCARNA10 | 0.90 | 0.68 | 2.30 (2^-△△Ct^) | 0.71 | 0.77 | 0.86^*^ | 3.368 | < 0.01 |
| AFP | 0.64 | 0.96 | 14.20 (ng/ml) | 0.97 | 0.70 | 0.84^*^ | 2.785 | < 0.01 |
| SCARNA10+AFP | 0.92 | 0.84 |  |  |  | 0.95 |  |  |
| **HCC vs BLD** |  |  |  |  |  |  |  |  |
| SCARNA10 | 0.74 | 0.80 | 2.30 (2^-△△Ct^) | 0.70 | 0.77 | 0.84^*^ | 3.262 | < 0.01 |
| AFP | 0.72 | 0.86 | 21.70 (ng/ml) | 0.93 | 0.69 | 0.78^*^ | 3.326 | < 0.01 |
| SCARNA10+AFP | 0.78 | 0.94 |  |  |  | 0.94 |  |  |

**p* < 0.01 in comparison with SCARNA10+AFP.
